# Hepatoprotective Effect of *Ficus carica* Leaf Extract on Mice Intoxicated with Carbon Tetrachloride

**Published:** 2011

**Authors:** Nasrin Aghel, Heibatollah Kalantari, Shohreh Rezazadeh

**Affiliations:** *Medicinal Plant Research Center, Joundishapour Medical Sciences University, Ahwaz, Iran.*

**Keywords:** *Ficus carica*, Liver, Carbon tetrachloride, Hepatoprotective, Mice

## Abstract

Protective action of *Ficus carica *leaf ethanolic extract (obtained by maceration) was evaluated in an animal model of hepatotoxicity induced by carbon tetrachloride (CCl_4_).

Male albino mice were divided into six groups. group I was normal control group; group II received olive oil (CCl_4_ solvent), groups III-VI received CCl_4_. After inducing hepatic damage, group III served as control for CCl_4_; and groups IV- VI received different doses of *Ficus carica *ethanol extract (200, 400 and 800 mg/kg) prior to intoxication with CCl_4_. Liver marker enzymes were assayed in serum. Sections of livers were observed under microscope for the histopathological changes. Levels of marker enzymes such as alanine aminotransferase (ALT) and aspartate aminotransferase (AST) were increased significantly in CCl_4_ treated mice (group III). In groups IV, V and VI, pre-treated with the plant extract and intoxicated with CCl_4_, decreased activities of these two enzymes were observed. Also, pre-treatment with the extract in these groups resulted in less pronounced destruction of the liver architecture with no fibrosis and moderate inflammation was observed compared with group III. The present observations suggested that the treatment with *Ficus carica *leaf extract in dose of 200 mg/kg enhanced protection against CCl_4_ induced hepatic damage.

## Introduction

The genus *Ficus *belongs to the family Moraceae and to the order urticales (1). Moraceae is composed of trees and shrubs which characteristically have a milky juice. *Ficus *constitute one of the largest genera of angiosperms, with almost 800 species of terrestrial trees, shrubs, hemi-epiphytes, climbers and creepers occurring in the tropics and subtropics worldwide (2). *Ficus *are important genetic resources with high economic and nutritional value. They are also an important part of the biodiversity in the rainforest ecosystem by setting fruit throughout the year and providing an important source of food for fruit-eating animals in the tropics.

*Ficus carica *(fig tree) has been extensively investigated for its proteolytic enzymes (3), amino acids, minerals and sugars (4), triterpenes (5), and organic acids (6). The leaves are added to boiling water and used as a steam bath for painful or swollen piles, and as a decoction and stomachic (7). The fruit is mildly laxative, demulcent, digestive and pectoral (8). The leaf decoction is taken as a remedy for diabetes and calcifications in the kidneys and liver (9).

One of the traditional methods for treatment of warts in some rural areas of Iran is to use fig tree latex as a local treatment, as reported by Avicenna in his 10th century book *Canon of Medicine *(10).

Despite the fact that other parts of the fig tree, like the fig leaves, have also reported pharmacological properties, they have been much less investigated. In 1998, Serraclara *et al*. (11) reported the hypoglycemic action of a fig leaf decoction in type-I diabetic patients, and in 2000, Canal *et al*. (12) used a chloroform extract, obtained also from a decoction of *F. carica *leaves, to decrease the cholesterol levels of rats with diabetes. These pharmacological properties are probably in part due to the high content of phenolic compounds in these plant extracts. Administration of the *F. deltoidea *leaves aqueous extract produced significant dose-dependent antinociceptive effect in animal model (13).

Liver is the first major organ to be exposed to ingested toxins due to its portal blood supply and toxins may be, at least partially, removed from the circulation during the first pass, providing protection to other organs while increasing the likelihood of hepatic injury (14). Liver toxicity is monitored in standard toxicity studies by a range of investigations including clinical biochemistry parameters (enzymes, proteins, lipids, *etc.*). The following endpoints are considered to be mainly related to liver toxicity: its relative weight and more than two enzymes indicative of hepatocellular effects such as (alanine aminotransferase, aspartate aminotransferase, alkaline phosphatase and lactate dehydrogenase) (15).

Unfortunately, conventional or synthetic drugs used in the treatment of liver diseases are inadequate and sometimes can have serious side effects. This is one of the reasons for many people in the world over including those in developed countries turning complimentary and alternative medicine. Many traditional remedies employ herbal drugs for the treatment of liver ailments (16). A number of plants have been shown to possess hepatoprotective property (17-19).

Considering that other species of *Ficus *had hepatoprotective activity, the present investigation was undertaken to test the efficacy of different doses of ethanolic extract prepared from the leaves of *Ficus carica *against hepatic injury induced by CCl_4_ in mice to determine the possible use of this plant in preventing hepatic damage.

## Experimental

*Materials and methods*

*Animals*

Male albino mice weighing 30-31.5 g were obtained from Animal House of Ahwaz Joundishapour University of Medical Sciences and were kept at 23-25°C, under the light and dark cycles of 12 h, at 60-80% relative humidity, and fed with a standard diet and watered *ad libidum*. All experiments were performed in the morning.

*Chemicals*

Assay kits for the estimation of serum enzymes (ALT and AST) were purchased from pars azmun (liquid test). All other chemicals were of analytical grade.

*Plant material*

Leaves of *Ficus carica *were collected from campus garden of School of Pharmacy in summer of 2007. The plant material was identified and authenticated taxonomically by Dr. Sedigi, Faculty of Agriculture, Shahid Chamran University, Ahwaz, Iran and a voucher specimen was stored in the herbarium of Pharmacognosy Department, School of Pharmacy, Ahwaz Joundishapour University of Medical Sciences, Ahwaz, Iran.

*Preparation of plant extract*

The leaves were shade dried, powdered and then were extracted with 80% aqueous EtOH by maceration at room temperature for 72 h. The extract was filtered and the filtrate was concentrated in a rotary evaporator under reduced pressure.

*CCl*_4_ *induced hepatotoxicity*

Mice were divided into six groups of seven animals in each group. Table 1 shows the dose schedule of carbon tetrachloride and test samples against CCl_4_ intoxication. Each mouse was treated for four days with saline (0.9%), olive oil (CCl_4_ solvent); CCl_4_ (0.2 mL/kg in olive oil) or test samples (plant extract) orally in the total volume of 0.2 mL. Mice in test groups, in the second and third day, were given CCl_4_ half an hour after the administration of the plant extract dose. Liver weight changes, biochemical and histopathological evaluation were undertaken on the fifth day.

**Table 1:** Dose schedule

**Groups**	**Days**				
	1	2	3	4	5
(I) Negative Control	Normal Saline (0.9٪)	Normal Saline (0.9٪)	Normal Saline (0.9٪)	Normal Saline (0.9٪)	Sacrifice
(II) Olive Oil	Normal Saline	Olive Oil	Olive Oil	Normal Saline	Sacrifice
(III) CCl_4_	Normal Saline	CCl_4_	CCl_4_	Normal Saline	Sacrifice
(IV) Test 1	200 mg/kg	200 mg/kg + CCl_4_	200 mg/kg + CCl_4_	200 mg/kg	Sacrifice
(V) Test 2	400 mg/kg	400 mg/kg + CCl_4_	400 mg/kg + CCl_4_	400 mg/kg	Sacrifice
(VI) Test 3	800 mg/kg	800 mg/kg + CCl_4_	800 mg/kg + CCl_4_	800 mg/kg	Sacrifice

Negative control received normal saline, Test 1, 2 and 3 received 200, 400 and 800 mg/kg of *Ficus carica *leaf extract, respectively

Animals were killed in the fifth day by using chloroform, their blood was collected, allowed to clot and serum was separated by centrifugation at 3000 rpm for 15 min. Liver was dissected out and used for histopathological studies.

*Biochemical determination*

The biochemical parameters (serum enzymes): alanine aminotransferase (ALT) and aspartate aminotransferase (AST) were assayed spectrophotometrically using a commercially available assay kits according to the manufacturer’s protocol.

*Liver histopathological assessment*

Liver sections taken immediately from liver, fixed in 10٪ formalin, dehydrated in gradual ethanol (50-100٪), cleared in xylene and embedded in paraffin. Sections (4-5 μm thick) were prepared and then stained with hematoxylin and eosin (H-E) dye for photomicroscopic observation, including cell necrosis, fatty change, hyaline degeneration, ballooning degeneration, infiltration of Kupffer cells and lymphocytes.

*Statistical analysis*

The data are expressed as mean ± SD. Data were analyzed by analysis of variance (ANOVA) followed by multiple comparisons using Tuky test to compare all groups against control. Results were considered statistically significant at p < 0.05.

## Results and Discussion

Liver is a major site for metabolism of exogenous chemicals (pesticides, drugs, metals), resulting in the formation of metabolites which may be more or less toxic than the parent compound (20). CCl_4_, an extensively studied liver toxicant, and its metabolites such as CCl_3_ radicals are known to be involved in the pathogenesis of liver damage.

The yield (w/w) of the ethanolic extract was 6.36%. No mortality observed in any of the groups. The results of hepatoprotective effects of different *Ficus carica *leaf extracts on CCl_4_ intoxicated mice are shown in Table 2. There was no significant changes in the activities of serum ALT, AST and liver weight in group of olive oil mice compared to negative control group (Table 2). The liver weights of the three Test groups calculated at the end of the study had decreased (statistically significant) when compared with CCl_4_ induced group (Table 2). In the three test groups the level of marker enzymes were found retrieving towards normalcy.

**Table 2:** Effect of different *Ficus carica *leaf extract on CCl_4_ induced rise in serum ALT, AST and changes in liver weight in mice

**Groups (n = 7)**	**ALT (IU/L) ( Mean ± SEM)**	**AST (IU/L) ( Mean ± SEM)**	**Liver weight (g) ( Mean ± SEM)**
Negative Control	116.75 ± 1.60	138.15 ± 5.05	1.60± 0.035
Olive Oil	117.14 ± 2.99	145.28 ± 2.95	1.41 ± 0.025
CCl_4_	190.90 ± 1.35^+^	239.40 ± 2.84^+^	1.89 ± 0.079^+^
Test 1	85.57 ± 4.23*	194.71 ± 9.88*	1.22 ± 0.074*
Test 2	96.71 ± 2.99*	204.00 ± 14.86*	1.50 ± 0.084*
Test 3	132.43 ± 15.76*	224.00 ± 11.21	1.50 ± 0.081*

Negative control received normal saline, Test 1, 2 and 3 received 200, 400 and 800 mg/kg of *Ficus carica *leaf extract, respectively. p* < 0.05 as compared with CCl_4_ induced group. p^+^ < 0.05 as compared with negative control group

**Figure 1:**
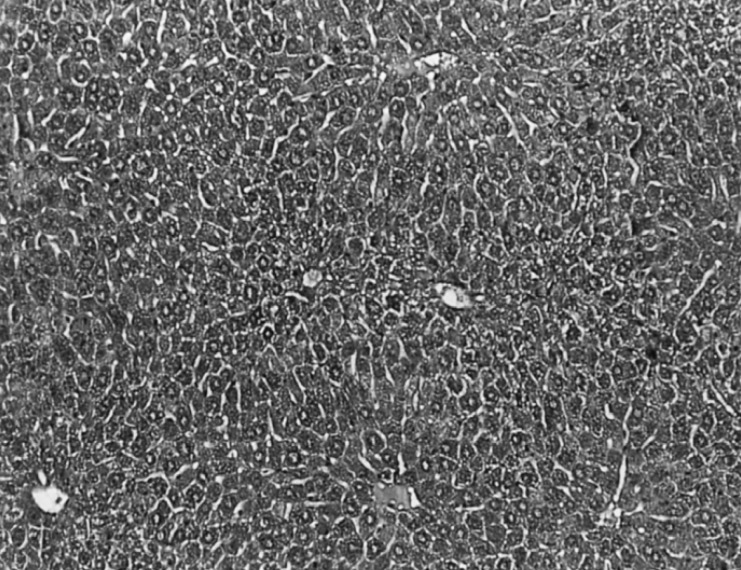
Light microphotographs of HE- stained sections (100X) of the formalin fixed liver of normal control group showing normal hepatic architecture

The histopathological observation basically support the results obtained from enzyme assays. Histology study of liver from negative control group showed a normal hepatic architecture (Figure 1). 

**Figure 2:**
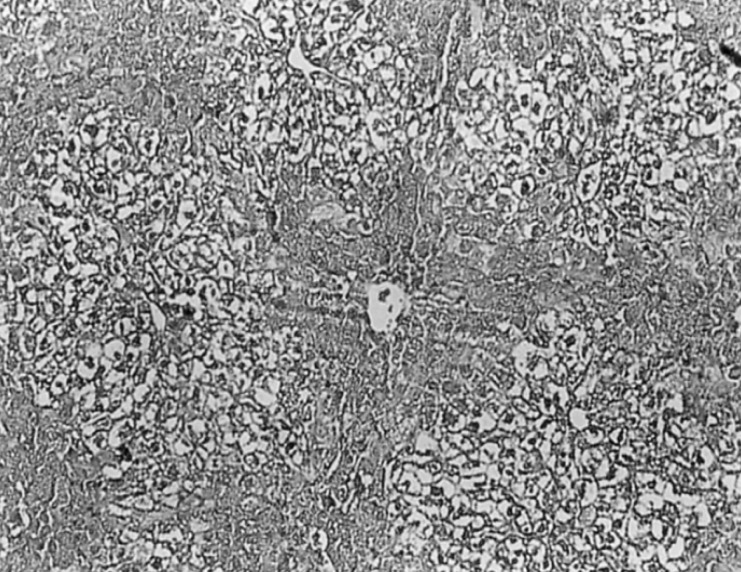
Light microphotographs of HE- stained sections (100X) of the formalin fixed liver of carbon tetrachloride control group showing severe hepatotoxicity

In CCl_4_ induced group, severe hepatotoxicity (massive fatty changes, necrosis, ballooning degeneration and broad infiltration of the lymphocytes and Kupffer cells around the central vein) was seen (Figure 2). 

**Figure 3:**
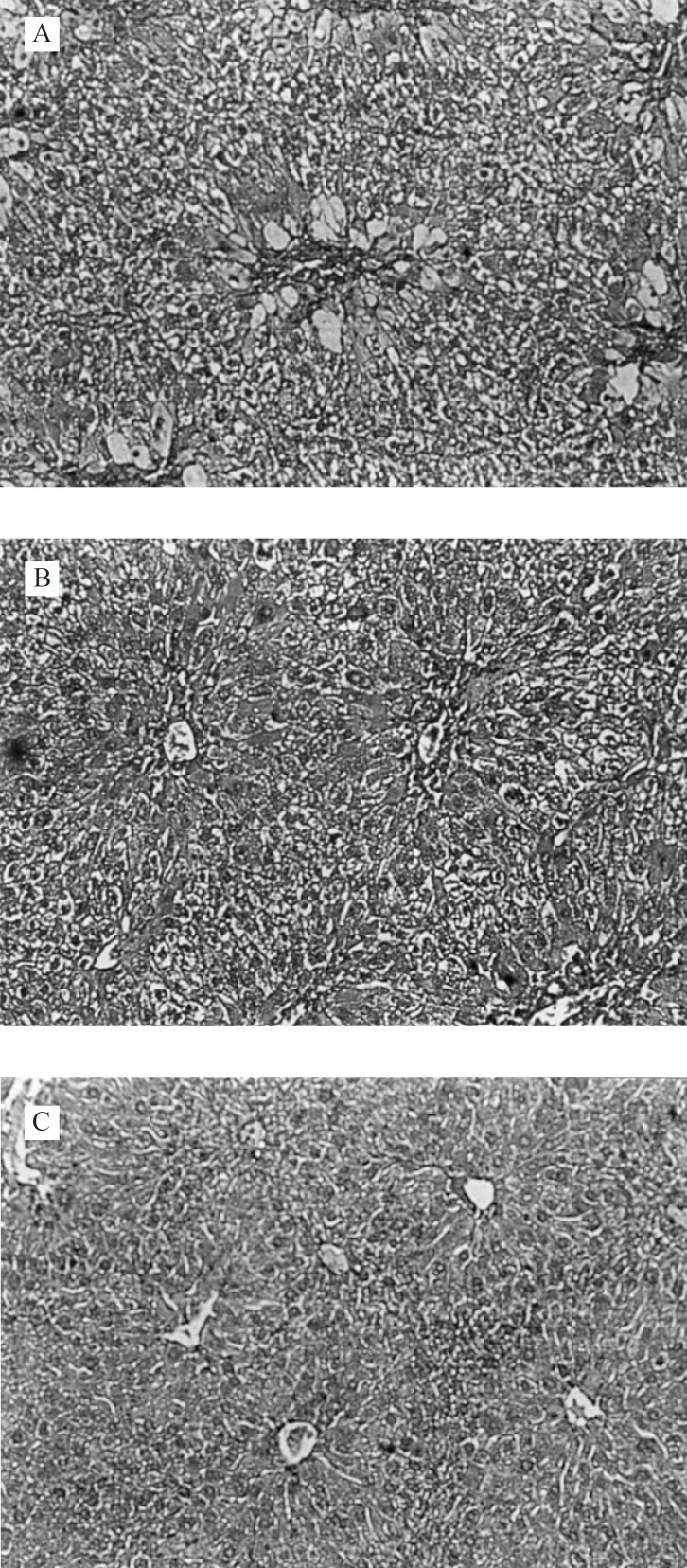
Light microphotographs of HE- stained sections (100 X) of the formalin fixed liver of Ficus carica leaf extract pre-treated groups: A (200 mg/kg, p.o.); B (400 mg/kg, p.o.) and C (800 mg/kg, p.o.); showing a more or less normal lobular pattern

The histological architectusre of liver sections of mice treated with different *Ficus carica *leaf extracts showed a more or less normal lobular pattern with a mild degree of fatty change, necrosis and lymphocyte infiltration almost comparable to the normal control group (Figure 3). 

In previous investigations , hepatoprotective effect of leaf extracts of other species of *Ficus* were examined. Mandal *et al. *showed that an oral dose of 400 mg/kg of *Ficus hispida* methanolic leaf extract exhibited a significant protective effect against paracetamol-induced hepatotoxicity in rats (21). The hepatoprotective activity of *Ficus racemosa *leaf extract on liver damage caused by carbon tetrachloride in rats was comparable to standard liver tonic (22). 

In another study, carried out by Krishna Mohan *et al*., the protective effect of methanolic

leaves extract of *Ficus carica *was reported at a dose of 500 mg/kg (23). In the present research, we tried three different doses and obtained a significant liver protection at dose of 200 mg/kg.
